# The Book World of Medicine and Science

**Published:** 1905-10-14

**Authors:** 


### The Book World of Medicine and Science.

A Plea for the More Energetic Treatment of the Insane. By Charles Williams, L.R.C.P., etc. (Pamphlet, pp. 51. Price 1s. 6d. net.)

It can hardly be said that this pamphlet contains anything particularly striking or particularly new. Its title sufficiently explains its purport. It advocates the resuscitation of some old forms of treating the insane and the persistence in some newer forms; and, if we gather the meaning rightly, that such treatment should be tried with classes of the insane rather than with individuals. Nothing is more certain than that a considerable—nay, a high relative—percentage of recoveries will take place in the ordinary run of cases admitted to our county asylums under almost any kind of treatment that is not prejudicial; and at the opposite pole there is a certain proportion doomed from the very beginning to end in some incurable form of brain disease. But there is a section between these two, and it is presumably to this section that Dr. Williams would have his "energetic" treatment directed. Certain it is that recovery in isolated cases will not carry conviction of the value of any particular line of treatment, for who is there among us who cannot recall numerous instances of recovery in the (apparently) chronic insane when the treatment was merely dietetic and hygienic? Counter-irritation is one of the weapons Dr. Williams would awaken from its peaceful sleep; and it may be that recovery would thereby be obtained in rare instances, or hastened in others, by "severe, deep-seated, and long-continued" counter-irritation as the author recommends; but, so far as we can see, there are no means whereby we could distinguish beforehand between those who would be benefited and those who would be injured; and would Dr. Williams advise that all patients in a particular class should be subjected to this heroic treatment for the possible, or probable, good in one case? If he did advise, it is possible that he would find it a little difficult to convert even one asylum medical officer to his views. The pamphlet takes us over a wide range. The electric treatment, prolonged warm bath, animal extracts, argument, suggestion, and subterfuge are spoken of. As regards the last, Dr. Williams recommends that in the case, say, of a man who believed he had no stomach, that a sort of sham operation might be performed and a sham stomach brought into misty outline; and these, with a whiff of chloroform, might dispel the delusion. Dr. Williams admits that the delusion is not the insanity, and, if it were banished, the insanity would remain; but even then he would approve of the step, because the delusion is very real to the patient and causes him much suffering. But we may ask what security have we that the old delusion will not be followed by a new and a worse one? There is, however, much in the pamphlet to make one think, and that is no bad quality in any publication.

"Clinical Methods." By Robert Hutchison, M.D., F.R.C.P., and Harry Rainy, M.A., F.R.C.P.Ed. Third edition. (London: Cassell and Company, Limited. 1905. Pp. 634. Price 10s. 6d.)

We gladly welcome a new edition of this well-known and extremely useful book. Pathologists are very active just now, and are constantly suggesting novel methods of clinical examination, for use by the bedside as well as in the laboratory; and it is by no means easy for the medical practitioner either to appraise the real merits of these innovations or to learn their use when he has satisfied himself as to their value. But he will be greatly aided in solving these difficulties by reference to "Clinical Methods." The authors have made a special point of including all such modern advances as appear likely to prove of permanent value and are capable of clinical application. The result is an unqualified success, and we cordially recommend this new edition to the attention of medical men.

"A Manual of Surgery for Students and Practitioners." By William Rose, M.B., B.S.Lond., F.R.C.S., and Albert Carless, M.S.Lond., F.R.C.S. Sixth edition. (London: Baillière, Tindall, and Cox. 1905. Pp. 1350. Price 21s. net.)

Little more need be said about this new and thoroughly revised edition than to report its issue. As a text-book of surgery the work is sufficiently well known to render general comment unnecessary. The opening chapter on "Bacteriology" has been rewritten by Dr. W. D'Este Emery, who has provided also a new chapter on "The Blood in Health and Disease." A fresh chapter has been devoted to modern surgical technique, and another dealing with the surgical aspects of gynæcology has been added. Although reference is made to the *x*-rays in various parts of the book, and their uses and limitations in certain conditions explained, we think it would have been advantageous to include a separate chapter on this subject alone. On the whole the new edition of this manual of surgery is a distinct advance on previous editions, both as regards the quantity and the quality of the material it contains.

"The Doctor Says." (London: Sidney Appleton. 1905. Pp. 306. Price 3s. 6d. net.)

One of the proper functions of the medical profession is to point out to the public how illness is to be avoided. Another important function is to teach people to appreciate the earliest symptoms of illness, so that they may not lose time before resorting to suitable remedies or seeking the help of their medical advisor. Both of these offices are admirably embodied in the book before us. "The Doctor Says" is a medical text-book written for laymen, and the writer is evidently an individual who can speak with authority on the matters with which the book deals. There is little room for fault-finding in the substance of the advice which is given or the manner in which it is expressed. Purely medical expressions are eliminated so far as possible, and the text is therefore quite easy to follow. We do not think it is going too far to say that every householder would be well repaid if he were to get this book and carefully read, mark, learn, and inwardly digest all that "The Doctor Says."

The Rational Treatment of Running Ears. By F. Faulder White, F.R.C.S.Eng. First Edition. (London: Iliffe and Sons. 1905. Price 5s.)

This little book has been written to show what good results can be attained by the operation of otectomy in the treatment of obstinate otorrhœa. Mr. White is an earnest advocate for a more conservative treatment of suppurative otitis than the radical operation so frequently performed at the present time. He states his method clearly, and gives details of a number of cases successfully treated. There is much that is of value in Mr. White's work, although one cannot agree with him when he says that on the whole the radical operation has done more harm than good to sufferers from suppurative otitis.

"Where Shall I Send My Patient?" (Bournemouth: The Association of Medical Men receiving Resident Patients.)

This little book is intended to serve as a guide to medical practitioners as to where to send their patients. It gives a list of houses for resident patients, asylums, convalescent homes, etc., but the main intention naturally is to give publicity to the accommodation offered to patients in the residences of the members of the Association. The names and addresses are not given, but the accommodation and locality are described, and the names are revealed on application to the hon. secretary. It is surprising to learn that the public can be received by about 126 medical men, who are ready to treat almost every description of case. The book is well got up, and contains some useful information concerning health resorts, which is arranged in a simple and practical manner.

